# Understanding the Role of Galectin-1 in Heart Failure: A Comprehensive Narrative Review

**DOI:** 10.2174/011573403X274886231227111902

**Published:** 2024-01-08

**Authors:** Mohammadjavad Sotoudeheian, Seyed-Mohamad-Sadegh Mirahmadi, Mohammad Pirhayati, Reza Azarbad, Soroush Nematollahi, Mehdi Taghizadeh, Hamidreza Pazoki-Toroudi

**Affiliations:** 1 Physiology Research Center, Department of Physiology, Faculty of Medicine, Iran University of Medical Sciences, Tehran, Iran;; 2 Department of General Medicine, School of Medicine, Iran University of Medical Sciences, Tehran, Iran;; 3 Cellular and Molecular Biology Research Center, Health Research Institute, Babol University of Medical Sciences, Babol, Iran;; 4 Faculty of Medicine, Iran University of Medical Sciences, Tehran, Iran;; 5 Cardiology Resident, Department of Cardiology, Shahid Madani Hospital, Tabriz University of Medical Sciences, Tabriz, Iran

**Keywords:** Cardiovascular disease, cardiomyocyte, MMP-9, inflammation, extracellular matrix, pathophysiology

## Abstract

Heart failure (HF) is the fastest-growing cardiovascular condition worldwide. The immune system may play a role in the development of HF since this condition is associated with elevated pro-inflammatory cytokine levels. HF is a life-threatening disease, and there is an increasing demand for diagnostic biomarkers, prognostic factors, and therapeutic agents that can help treat it. Galectin-1 (Gal-1) is the prototype galectin of the lectin family. Multiple signal transduction pathways are regulated by Ras proteins, which act as a molecular switch in cells. Gal-1 regulates T and B cell activation, differentiation, and survival. Gal-1 has been linked to inflammation. Activated T cells produce Gal-1 through an autocrine apoptotic mechanism involving MEK1/ERK and p38 MAPK. In the cardiovascular system, atherosclerosis is facilitated by Gal-1. Heart disease, myocardial infarction, hypertension, and stroke can be caused by atherosclerotic plaque. HF and heart hypertrophy are caused by decreased cardiac L-type Ca^2+^ channel activity. Deregulation of Gal-1 and Ca_V_1.2 in pathological cardiac hypertrophy suggests a possible target for anti-hypertrophic therapy. Rat hypertrophic cardiomyocytes express Gal-1 and Ca_V_1.2 channels simultaneously. It has been reported that diastolic dysfunction (DD) is associated with elevated Gal-1 levels. The high Gal-1 level in subjects led to the lowest cumulative survival as a composite endpoint. Incidences of HF, DD, and serum Gal-1 levels correlated significantly. The ejection fraction was negatively correlated with Gal-1 and CRP concentrations. Based on two different approaches in mice and humans, Gal-1 was identified as a potential mediator of HF.

## 1 INTRODUCTION

Several cardiovascular conditions are experiencing rapid growth worldwide, but heart failure (HF) is the fastest-growing [1]. There is still an increase in the number of HF patients today due to the aging population [2, 3]. A growing proportion of HF patients in developed countries have preserved ejection fractions, and improved therapies are needed [1]. Despite this, the mix of patients with HF is changing. Although the incidence has stabilized in some populations, it is likely to decrease in others due to a rise in obesity. These trends may cause alarming opposite trends among relatively young individuals. Further, the HF has clearly transitioned to an ejection fraction preserved HF [2]. The age-adjusted prevalence of HF in the developed world has decreased due to improvements in cardiovascular disease prevention and ischemic heart disease treatment [1].

Research has revealed a potential role of the immune system in the development of HF with reduced ejection fraction because of the observation that these conditions are associated with elevated levels of pro-inflammatory cytokines [4]. The evidence for chronic HF being perpetuated by inflammation is unclear. HF progression is associated with pro-inflammatory biomarkers, such as tumor necrosis factor, interleukin-1 (IL-1), interleukin-6 (IL-6), and galectin-3 (Gal-3) [2]. As compared to healthy individuals, patients with HF along with reduced ejection fraction (HFrEF) and New York Heart Association class II–IV have elevated Gal-3 levels in their plasma. There is evidence that Gal-3 is a promising prognostic marker for patients with HFrEF [4].

All organisms express galectins (Gals), a class of lectins. As a result of their sugar moieties, carbohydrates are recruited into signaling and adhesion networks that regulate cell communication, activation, adhesion, migration, and apoptosis. Inflammatory and immune cells are among the primary cells expressing Gals, which are expressed in different amounts by different cell types. Galectin family members are thought to regulate homeostasis and inflammation of immune cells. Since they are inflammation-related, they are attractive molecular targets in inflammation-associated diseases [5].

The increased number of HF patients encourages researchers to evaluate diagnostic biomarkers, prognostic factors, and therapeutic agents of HF. Previously, scholars have determined many biomarkers and suggested different molecular pathways to interfere in HF pathogenesis. As mentioned, Gal-3 is one of these biomarkers. In this review, we aimed to define galectin-1 (Gal-1). Therefore, we have critically discussed the role of this molecule in HF.

## 2 GALECTIN-1

Galectin-1 (Gal-1) is the prototype galectin of the lectin family, containing a distinctive carbohydrate recognition domain [6, 7]. As a molecular switch, Ras proteins are intracellular membrane-anchored proteins that regulate multiple signal transduction pathways [8]. A gene called LGALS1 encodes Gal-1 expression [7]. The secretion of this molecule is found in both normal and diseased tissues. A monomeric form is found mainly in the nucleus and cytosol [7]. Cell proliferation, cell cycle progression, pre-RNA splicing, and chemotaxis are affected by Gal-1 intracellularly, while adhesion, aggregation, migration, and chemotaxis are influenced by it extracellularly [5].

In the extracellular matrix, it enters through non-classical pathways [7, 9]. Cell growth, differentiation, signaling, and many other processes are regulated by it. Besides regulating the immune system, it also plays a vital role in regulating metabolism [7]. The redox environment [8] influences human Gal-1 extracellular and intracellular functions.

Most studies indicate that Gal-1 has anti-inflammatory and pro-resolving properties [5]. Many functions of the Gal-1 protein have been identified, including immune responses, cell growth, cell signaling, neoplastic transformation, and neovascularization [9, 10]. Moreover, the anticoagulant activity of FVIII can be influenced by Gal-1 binding, as demonstrated by O'Sullivan *et al.* [11].

As a negative-regulatory checkpoint on receptors required for signaling, Gal-1 regulates T and B cell activation, differentiation, and survival. Further, several studies have demonstrated that Gal-1 induction of apoptosis in T cells is mediated by Gal-1 [12]. Activated T cells undergo apoptosis when Gal-1 binds the CD45 receptor [5]. Similarly, anti-CD3 and Gal-1 stimulation enhances the production of IL-10 in peripheral blood mononuclear cells (PBMCs) compared to anti-CD3 stimulation alone. Furthermore, direct interactions between Gal-1 and CD45 promote the production of interleukin-10 (IL-10) by activated helper T cells.

Inflammation and cancer progression have been linked to Gal-1 [6]. In addition to increasing membrane anchorage and oncogenic H-Ras signaling, Gal-1 overexpression contributes to cell transformation. By stimulating vascular endothelial proliferation and migration, Gal-1 induces apoptosis in activated CD4+ and CD8+ T cells [6]. Programmed cell death is related to complex interactions of key regulators [13, 14]. As a result of Gal-1 treatment, both activated and non-activated CD4+ and CD8+ T cells produce more IL-10 mRNA and protein [12].

This suggests that Gal-1 is produced through an autocrine apoptotic mechanism operated by MEK1/ERK and p38 MAP kinase on activated T cells [5]. Baek *et al.* [6] showed that Gal-1 interacts with PPARγ and activates its transcription.

Consequently, Gal-1 plays a significant role in regulating immune function and preserving T cell homeostasis. Its expression is observed on both activated T cells and thymic stromal cells [15, 16]. Besides, Gal-1 acts as a negative-regulatory checkpoint on receptors required for signaling in B and T cells [5]. Gal-1 can be used as a treatment option for autoimmune conditions and other conditions characterized by inflammation [17]. Polymorphonuclear neutrophils are mainly affected by Gal-1's anti-inflammatory nature [17]. Gal-1 is related to maintaining neutrophil viability [18].

A Foxp3+ regulatory T cell (Treg) and a tolerogenic dendritic cell (DC) produce this substance [19-22], and it inhibits the immune system under both normal conditions and pathological conditions [23]. It contributes to the pro-resolving phenotype of macrophages through interferon γ (IFNγ)-mediated signalling by regulating the metabolism of L-arginine, which is responsible for their polarization toward an M2 profile [24, 25].

Gal-1 promotes a shift towards a T helper-2-dominant cytokine profile, which is beneficial in protecting against cardiovascular diseases. Gal-1 could be used as a therapeutic candidate to limit innate and adaptive responses during cardiovascular inflammation [26, 27]. Mice lacking Gal-1 showed adverse ventricular remodeling after myocardial infarction (MI), with increased cardiac dilation associated with dysregulated uncontrolled inflammation. The absence of Gal-1 led to an increase in cardiac infiltration by T lymphocytes, macrophages, and natural killer cells, while anti-inflammatory Treg cells were significantly reduced in this setting [26]. Gal-1 has been reported to control the Treg function [20, 28]. Gal-1 may also induce Treg-mediated protection during MI [26, 29].

Inflammation plays a critical role in the pathophysiology of many diseases [30-33], including cardiovascular events [34-36]. A pancreatic stellate cell that produces Gal-1 recruited inflammatory cells by causing proliferation and production of monocyte chemoattractant protein-1 (MCP-1) and cytokine-induced neutrophil chemoattractant-1 (CINC-1), activated by ERK, nuclear factor kappa B (NF-κB), and in part by JNK and ERK pathways [37, 38].

A positive correlation was found between serum Gal-1 and insulin resistance [6, 39, 40]. There has been a consistent correlation observed between LGALS1 and all genetic markers of lipid uptake and adipogenesis, as well as the markers of lipogenesis, adipocytes, and lipolysis [39]. The role of Gal-1 in regulating adipogenesis and fat accumulation has been studied. Adipose tissue and muscle are metabolic tissues that express Gal-1 mRNA highly [6]. As a result of Gal-1 silencing, some lipogenic factors were inhibited. They were inhibited either at mRNA or protein levels. Some of those lipogenic factors include PPARγ, C/EBPα, FABP4, and FASN [6]. A further study found that Gal-1 increases the risk of type 2 diabetes [41].

It is known that Gal-1 inhibits liver fibrosis, as well as hepatitis severity, and Gal-1 reduces heart inflammation after ischemia [42]. In addition, Gal-1 is believed to facilitate muscle regeneration in conditions of degenerative muscle and muscle damage [42]. The association between fibrotic markers [42] and *in vitro* studies is conflicting.

Atherosclerosis is facilitated by Gal-1 [43]. Several cardiovascular diseases can be caused by atherosclerotic plaque, such as coronary artery disease (CAD), myocardial infarction (MI), HF, and stroke [44]. In CAD, arteriogenesis can be associated with certain genetic variations. Galectin-2 gene polymorphisms, for example, have been linked to arteriogenic response in patients. Additionally, collateral artery formation can be influenced by polymorphisms in hypoxia-inducible factor 1-alpha (HIF-1α) [45]. The presence of numerous neutrophil polymorphs within the infarction center is accompanied by nuclear expression of HIF-1α. At 24 hours following MI, there was a notable abundance of neutrophil polymorphs within the infarction center. Additionally, these neutrophil polymorphs exhibited a significant level of expression of Gal-1 [46].

It has been shown that LGALS1 polymorphism is associated with other diseases, such as gestational diabetes and arthritis [47, 48]. Mice lacking Gal-1 had ventricular remodeling after myocardial infarction [26, 49]. The overexpression of Gal-1 led to an upregulation in the expression of vascular endothelial growth factor (VEGF) and VEGF receptors. Conversely, LGALS1 knockdown and gene polymorphism resulted in a downregulation of VEGF and VEGF receptor expression [50]. Moreover, polymorphism in the galectin genes could lead to atherothrombotic stroke [45]. According to He *et al.* [43], Gal-1 and Gal-9 levels increased following an ischemic stroke. There has been no association found between Gal-1 and Gal-9 and stroke prognosis in the short term [43].

When hypoxia occurs, HIF-1α is significantly up-regulated by PI3K/AKT and mitogen-activated protein kinase (MAPK)/ERK signaling. Intriguingly, HIF-1α and Gal-1 were found to be secreted by glial cells. This suggests a significant contribution of HIF-1α to the expression of this protein [37].

Studies involving humans and mice have shown that Gal-1 protects the kidney in conflicting ways [42]. Kuo *et al.* [9] reported that patients undergoing coronary angiography with higher serum Gal-1 levels are more likely to suffer from renal function declines. Drake and colleagues [41] reported a strong association between Gal-1 and lower glomerular filtration rate at baseline, but not with incident chronic kidney disease (CKD). However, cross-sectional and longitudinal studies have linked circulating Gal-1 to lower kidney function [42].

As discussed previously, Gal-1 is an important component of the immune system and plays a crucial role in autoimmune diseases. Experimental models have shown that Gal-1 expression peaks during recovery from autoimmune disease, suggesting that Gal-1 plays an essential role in inflammation resolution [19]. Inflammation is one of the numerous disease conditions that Gal-1 has been linked to due to its diverse activities [7]. The Gal-1 molecule suppresses the immune system, increases invasion and metastasis, and induces T-cell apoptosis in lung cancer [23]. During acute and chronic inflammatory responses, Gal-1 has been reported to play a wide variety of roles, and its therapeutic potential extends to a wide range of physiological and pathological diseases [19]. MI and congestive HF are known to be associated with altered Gal-1 regulation [42].

## 3 THE ROLE OF GALECTIN-1 IN HEART FAILURE

The increased number of HF patients encourages researchers to evaluate diagnostic biomarkers, prognostic factors, and therapeutic agents of HF. Previously, scholars have determined many biomarkers and suggested different molecular pathways to interfere in HF pathogenesis. As mentioned, Gal-3 is one of these biomarkers.

Human Gal-3 regulates a number of physiological and pathophysiological processes. Different types of tissues express Gal-3 differently, though tissue injury or stress can induce its expression. Studies have established an association between Gal-3 overexpression and secretion in various diseases, including fibrosis, HF, atherosclerosis, and diabetes [51, 52].

Several cardiovascular diseases have been reported to exhibit a double risk among people with type 2 diabetes, such as MI and stroke [42].

A number of studies have been conducted on the role of Gal-3 in physiological and pathological conditions. There has been a close relationship found between Gal-3, cardiac fibrosis, and HF based on several studies performed in the last decade involving healthy populations and HF patients. Guidelines recommended by the American Heart Association classify Gal-3 as class II in HF management. Furthermore, cardiac fibrosis and HF can be treated by targeting this molecule and its functional pathway [51].

In addition to Gal-3, Gal-1 and Gal-9 have also been shown to be critical molecules in regulating inflammatory responses, which makes Gal-3 not the only galectin that has potential biomedical applications [5]. Table **1** summarizes and compares the effects of Gal-1 and Gal-3.

The purpose of this paper was to review Gal-1’s mechanism of action in order to define its innate role in comorbidities related to the cardiovascular system, especially HF. Additionally, since different environmental factors, such as hypoxia, inflammation, aging, and metabolic status may affect Gal-1 expression, further work is needed to investigate their role in regulating its activity [26].

Several mechanisms and pathways are involved in Gal-1’s effects at the cellular level, whether they are physiological or pathological. Therefore, as extracellular Gal-1 acts to the same extent as the intracellular one, we, herein, initiate our discussion with the border of intra/extracellular space, the cellular membrane.

Cardiomyocyte Ca_V_1.2 channels allow Ca^2+^ influx through their lipid membranes. During cardiac stress states, Ca^2+^-induced Ca^2+^ release initiates excitation-contraction coupling. HF and cardiac hypertrophy are associated with altered Ca_V_1.2 calcium channel activity [53]. Heart hypertrophy and HF are caused when cardiac L-type Ca^2+^ channel activity is decreased [54]. Hypertrophic cardiomyocytes and hearts from rats express Gal-1 and Ca_V_1.2 channels synchronously [55]. The dysregulation of Gal-1 and Ca_V_1.2 suggests a possible anti-hypertrophic target in the heart in pathological cardiac hypertrophy [55]. Gal-1 has also been shown to affect calcium channel function in L-type Ca_V_1.2 [53]. Activating calcium/calmodulin-dependent protein kinase II via Gal-1 inhibits cardiomyocyte hypertrophy by reducing channel membrane expression [55].

Ca_V_1.2 calcium channels are degraded by Gal-1 binding directly to I-II loops. Specifically, binding to the splice-variant led to a decrease in the Ca_V_1.2 calcium channel current, regulating blood pressure and vascular constriction. In mouse and human acute MI, Gal-1 expression was increased, and Gal-1 may play a role in ventricular remodeling. Regardless, it is largely unknown whether Gal-1 affects cardiac Ca_V_1.2 calcium channel activity and cardiomyocyte hypertrophy in any way [55].

It has also been observed that Gal-1 increases TNF-α-mediated inflammatory responses. As a result of TNF-α-mediated activation of ERK1/2 signaling, MMP9 is induced in fibroblasts. Previous studies have shown that Gal-1 activates downstream Raf-1/MAPK signaling by interacting with Ras to enhance its membrane anchorage [56]. Fig. (**1**) summarizes the molecular pathways contributing to Gal-1’s effects.

The involvement of Gal-1 in cardiac pathophysiology remains obscure despite substantial evidence of its role in inflammation, immunoregulation, immunopathology, and angiogenesis [49, 57]. The absence of Gal-1 resulted in increased cardiac infiltration by T lymphocytes, macrophages, and NK cells, and a reduction in immunosuppressive Tregs [24]. An increase in lymphocytes, macrophages, and NK cells, as well as a reduction in Treg cells, were observed in mice lacking Gal-1, consistent with autoimmune myocarditis [26]. Moreover, LGALS1 knockout mice produced higher levels of circulating Th1 and Th17 cytokines, which might contribute to ventricular dysfunction and dilation, similar to inflammation-induced dysfunction induced by IL-1β and IL-18 [26].

Several studies have evaluated Gal-1's role in the cardiovascular system. Ou and colleagues [58] found that rats treated with Gal-1 had a significant reduction in inflammatory factors in their myocardium and serum after they were treated with the compound. Based on these findings, Gal-1 appears to enhance heart function in rats [58]. Likewise, Chiang *et al.* [56] investigated the role of Gal-1 in vascular diseases. They demonstrated that oxidized Gal-1 activated the MAPK pathway in macrophages, smooth muscle cells, and fibroblasts, which in turn induced the expression of MMP9 and inflammatory cytokines [56].

Gal-1 has also been implicated in the pathogenesis of hypertension (HTN) based on increasing studies in smooth muscles [53]. It is well known that chronic HTN eventually leads to HF. As a result, the majority of patients with HF have had a history of HTN at some point [59].

It is believed that extracellular vascular endothelial growth factor receptor-2 (VEGFR-2) cleavage contributes to the pathogenesis of HTN [60]. In vascular endothelial proliferation and apoptosis, VEGFR-2 plays a key role. Capillaries undergo apoptosis when VEGFR-2 signaling is depleted [60].

The binding of Gal-1 to neuropilin-1 (NRP-1) induced VEGFR-2 signaling and facilitated the proliferation, adhesion, and migration of endothelial cells [45]. Additionally, Gal-1 bound to CD146 on endothelial cells, preventing apoptosis and enhancing angiogenesis [45]. Gal-1 is mainly expressed by endothelial cells and cardiomyocytes in normal hearts. Compared to the left ventricle, the right ventricle exhibits more pronounced expressions. However, some studies have shown that most endothelial cells and a few cardiomyocytes express Gal-1 in the left ventricle [61].

Further, inflammation induces fibroblasts, endothelial cells, and smooth muscle cells to respond to inflammatory cytokines. Under ischemic conditions, endothelial cells and leukocytes consistently express MMP9 [60]. Unlike serum Gal-1 levels in coronary artery disease, serum MMP9 levels in coronary artery disease patients were significantly higher [56].

In an experimental study, scholars showed that Gal-1 prevented pathological vascular remodeling in atherosclerosis [57]. Atherosclerosis can lead to MI and HF as the atherosclerotic plaque progresses [44]. During acute MI, Gal-1 might play a protective role by decreasing inflammation and preventing remodeling [45]. Increased cardiac dilation in Gal-1-deficient mice was associated with uncontrolled inflammation and myocardial remodeling after acute MI [24].

Gal-1 has been shown to colocalize with sarcomeric actin on I bands in the cytosolic compartments of cardiomyocytes [44, 49]. Furthermore, Gal-1 has been reported to be significantly up-regulated in inflammatory microenvironments and essential in hypoxia-regulated transcriptomes, suggesting that Gal-1 might play a role in post-acute MI ventricular remodeling and HF [49]. In patients suffering from an acute MI resulting in adverse ventricular remodeling, the risk of developing HF has been found to be increased [26]. Post-MI ventricular remodeling and HF are thought to be mediated by Gal-1 [44].

Furthermore, the hypoxia-regulated transcriptome includes Gal-1, which is highly expressed in inflammatory microenvironments [44]. In hypoxic/reoxygenated cardiomyocytes, Gal-1 inhibited inflammation and apoptosis [58]. Inflammatory cytokines are produced during myocardial ischemia/reperfusion injury (MI/RI), including IL-1, IL-6, and TNF-α. Inflammatory cells are induced to infiltrate the myocardium [58]. As a result of pro-inflammatory cytokines, cardiomyocytes increase Gal-1 production *in vitro*. In mice with MI and patients with advanced HF, Gal-1 expression was elevated in heart tissues [44].

An animal model for MI showed Gal-1 and HIF-1α to be coexpressed in cardiac diseases. Ischemic myocardium expresses HIF-1α, which regulates angiogenesis [45]. The presence of Gal-1 reduced inflammation and apoptosis in cardiomyocytes after ischemia-reperfusion in the myocardium [58].

Seropian *et al.* [49] documented that mice lacking Gal-1 exhibited enhanced cardiac inflammation. Therefore, their results indicated that Gal-1 prevented inflammation in the heart during normal cardiac homeostasis and remodeling after an infarction.

Likewise, studies have shown a significant increase in left ventricular Gal-1 at 30 minutes post-MI. At 4 hours following MI [46], plasma Gal-1 levels increased significantly as well. The level of Gal-1 in the left ventricle increased in the early stages of ischemic tissue damage. Also, after 20 minutes of MI, HIF-1 α also significantly increased. In addition, the serum Gal-1 level of mice was reported to be raised as early as 4 hours post-MI [46]. HF levels and mortality are strongly correlated among patients with MI [62]. A study was conducted on patients with advanced HF to examine Gal-1 expression since Gal-1 is an essential regulator of immune responses [26]. HF can thus be attenuated in acute MI by Gal-1 treatment [49].

Gal-1 may attenuate cardiomyocyte apoptosis after MI/RI. Gal-1 treatment reduced bax and Bcl-2 mRNA levels, as well as caspase-3 and caspase-8 mRNA. MI/RI significantly increased the rate of cardiomyocyte apoptosis compared to control, and Gal-1 treatment significantly reduced that rate [58].

A comparison of explanted hearts of patients with HF undergoing transplantation with those of healthy controls revealed an increase in Gal-1 expression. Gal-1 is located within the infiltrate and interstitium of the inflammatory process, as well as in cardiomyocytes [26]. A homeostatic mechanism may control the onset of autoimmune myocarditis in patients with HF by increasing Gal-1 expression. Cardiomyopathy and HF may be caused by intracellular Gal-1 based on its role in cardiomyocyte contraction [26].

Wassenaar *et al.* [63] documented LGALS1 to have a significant *p*-value of 0.02 and a high beta coefficient (0.32), corresponding to an odds ratio of 1.38 for HFpEF. A new potential mediator in the development of HFpEF was identified using two different approaches in mice and humans, and Gal-1 was identified as a potential candidate [63, 64].

It has been found that HF and mortality are associated with diastolic dysfunction of the left heart (DD). An investigation of the associations between serum Gal-1 levels, DD, and HFpEF was conducted in a clinical study. Gal-1 levels were associated with higher left ventricular mass indexes, left atrial diameters, and DD prevalence in patients. The composite endpoint was also significantly linked to elevated levels of Gal-1. A significant association between elevated Gal-1 levels and DD remained after adjusting for confounding factors. A composite endpoint was observed with the lowest cumulative survival in the highest Gal-1 group. There was a significant correlation found between serum Gal-1 levels and all-cause mortality, incident HFpEF, and DD [65].

There was a significantly higher prevalence of DD found among subjects in higher Gal-1 groups. DD is independently associated with high Gal-1 levels. Gal-1, high-sensitivity C-reactive protein (hs-CRP), and NT-proBNP exhibit significant correlations, providing indirect evidence that chronic inflammation and elevated left atrial pressure are related [65]. LVEF and hemoglobin concentrations were negatively correlated with Gal-1 and hs-CRP concentrations, whereas WBC count and fasting glucose were positively correlated with both of those concentrations [44].

It seems that a higher level of Gal-1 concentration is associated with an aging population, HTN, diabetes, CKD, HF, and a higher level of hs-CRP. A number of factors have been identified as prognostic factors for major adverse cardiovascular events (MACEs), such as age, gender, comorbidities, co-medications, serum levels of hemoglobin, creatinine, hs-CRP, ejection fraction, and methods of revascularization. However, patients in the highest tertile of Gal-1 were also more likely to suffer from MACEs than those in lower tertiles [44].

## CONCLUSION

In conclusion, several studies on Gal-1 have highlighted the unique role of this lectin in the pathophysiological mechanisms that lead to diseases. As a result of the effects of this molecule on cardiomyocytes and the regulation of the events that contribute to HF, the molecular bases of HF occurring due to this lectin have yet to be thoroughly studied in detail. The diagnostic ability, the prognostic threshold, and the therapeutic role of this lectin remain to be studied in much more detail. Due to this fact, it is significant to note that although there have been studies conducted on the role of Gal-1 in cardiovascular diseases, especially HF, there are currently no clinical trials to confirm its prognostic value, molecular effects, and therapeutic properties. A more detailed study of the molecular mechanism and pathways involved in the action of Gal-1 would be necessary to clarify the impact of this molecule.

## Figures and Tables

**Fig. (1):**
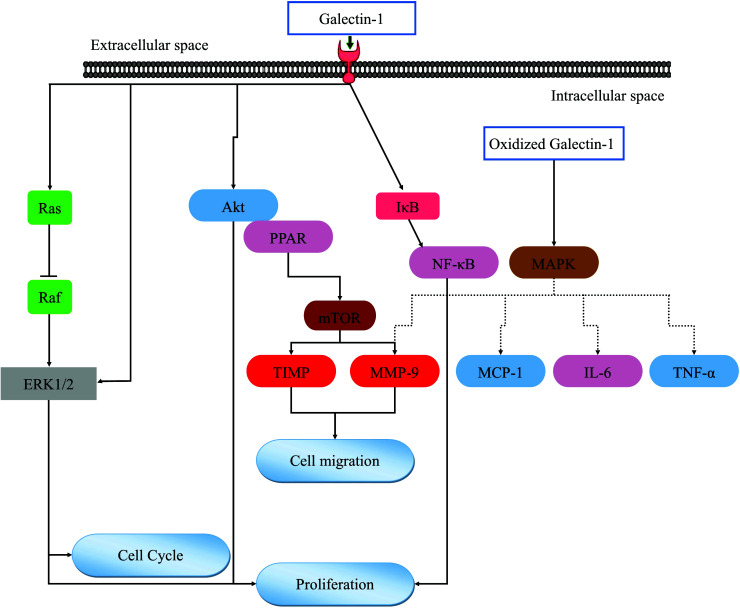
The molecular pathways influenced by Gal-1.

**Table 1:** A brief review on galectins and a comparison between galectin-1 and galectin-3.

-	Galectin-1	Galectin-3
**Angiogenesis**	• Increased EC proliferation • Increased EC migration • Increased EC adhesion • Increased EC survival • Binds NRP-1 activating VEGFR • Binds CD146 on ECs • EC apoptosis • Modulation of SMCs • Co-expression with HIF-1α	• Increased EC proliferation • Increased EC migration • Increased M2-macrophage infiltration • Platelet activation causing VEGF release • Transmembrane signaling via ɑ3β1
**Arteriogenesis**	• Increased monocyte migration • Increased NRP migration	• Binds to macrophages • Polarization of macrophages
**Atherosclerosis and myocarditis**	• Increased SMC proliferation • Increased SMC adhesion to ECM • Increased LPA accumulation	• Secretion by plaque foam cells • Attraction of monocytes/macrophages • Increased intracellular cholesterol accumulation • Transforming macrophages to foam cells • Binds LPS • Increased expression in M2 macrophages • Increased expression in myocarditis (macrophages) • ↓ myocarditis severity after blockage of Gal-3
**Myocardial infarction and ischemic stroke**	• Positively associated with neurogenesis • Associated with better outcome after ischemic stroke • Anti-inflammatory effect after MI	• Increased expression 30 h after STEMI • Decreased levels > reperfusion < 3h in STEMI • Role in the recovery period after STEMI • Proliferation in cerebral ECs, microglia, and progenitor cells • Mediation of VEGF and MIF
**Heart failure**	• Anti-inflammatory effect • After MI, protecting myocardium from remodeling	• Promotes macrophages • Increased mast cell infiltration • Increased fibroblast proliferation • Increased adverse cardiac remodeling/fibrosis/collagen deposition • TGF-β/Smad3 signaling • Acts as a biomarker of heart failure

**Note:** [45]

**Abbreviations:** ECM: extracellular matrix, EC: endothelial cells, NRP-1: neuropilin-1, VEGFR-2: vascular endothelial growth factor receptor-2, SMC: smooth muscle cells, HIF-1 α: hypoxia-inducible factor-1 alpha, LPA: lipoprotein a, LPS: lipopolysaccharide, STEMI: ST-elevation myocardial infarction, MIF: migration inhibitory factor, TGF: transforming growth factor.

## LIST OF ABBREVIATIONS

DD: Diastolic Dysfunction
HF: Heart Failure
PBMCs: Peripheral Blood Mononuclear Cells
MI: Myocardial Infarction
